# 

**Published:** 1850-03

**Authors:** 


### CONTENTS:

PART I.—ORIGINAL COMMUNICATIONS.

ART. 1.—Therapeutic Use of Hygienic Measures—By Prof. Jno. McLean, M.D. - - - - - - - 462
ART. 2.—Two Cases of Lead Colic—By R. A. Spencer, M.D., - 467
ART, 3.—A Case of Eclampsia Parturientium—By David Hutchinson, M. D., - - - - - - - - 468
ART. 4.—Guiacum in Dysmenorrhœa—By Dr. J. R. Stone. - 472
ART, 5,—Remarks on Medical Legislation—By E. M'Arthur, M.D., 475
ART. 6.—The Cholera, and the Influences which favor and retard its Spread or Diffusion—By Prof. N. S. Davis, M.D., - - 482
ART. 7.—Toxicology—By Wm. Nofsinger, M.D,, - - - 498
ART. 8.—Pneumonia Miasmatica—By J, J. Lescher, M.D., - 509
ART. 9.—Proceedings of the Fox-River Medical Society, - - 517

PART II.—REVIEWS AND NOTICES OF NEW WORKS.

ART. 1.—Proceedings of the North American Pomological Convention, - - - - - - - - - 519
ART. 2.—Thomson's Conspectus of the Pharmacopœias of the United States and Great Britain, - - - - - 522

PART III.—SELECTIONS.

ART. 1.—Abscess in the Substance of the Brain; the Lateral Ventricles opened by an Operation—By Wm. Detmold, M.D., - 523
ART. 2.—Trial of a Homœopathist for starving a patient to death, 533
ART. 3.—Treatment of Scarlatina by Inunction—By Dr. Schneeman, - - - - - - - - - 536

PART IV.—EDITORIAL.

ART. 1.—American Medical Association—Third Annual Meeting. 540
ART. 2.—New Medical Journals, - - - - - - 542
ART. 3.—State Medical Convention, - - - - - 544
ART. 4.—Our Journal, - - - - - - - 545
ART. 5.—Miscellany, - - - - - - - 546
ART. 6.—To Subscribers, - - - - - - - 548

